# Genomic and metabolomic insights into the antimicrobial compounds and plant growth-promoting potential of *Bacillus velezensis* Q-426

**DOI:** 10.1186/s12864-023-09662-1

**Published:** 2023-10-04

**Authors:** Lulu Wang, Ruochen Fan, Haodi Ma, Yu Sun, Yangzhu Huang, Yuxin Wang, Qinfeng Guo, Xinxiu Ren, Lukai Xu, Jing Zhao, Liying Zhang, Yongbin Xu, Liming Jin, Yuesheng Dong, Chunshan Quan

**Affiliations:** 1 School of Life Science and Biotechnology, Dalian University of Technology, No. 2 Linggong Road, Dalian, 116024 Liaoning China; 2 Department of Bioengineering, College of Life Science, Dalian Minzu University, Dalian, 116600 Liaoning China; 3 Key Laboratory of Biotechnology and Bioresources Utilization of Ministry of Education, College of Life Science, Dalian Minzu University, Dalian, China

**Keywords:** Genome mining, *Bacillus velezensis* Q-426, Secondary metabolites, Plant growth-promoting rhizobacteria

## Abstract

**Background:**

The Q-426 strain isolated from compost samples has excellent antifungal activities against a variety of plant pathogens. However, the complete genome of Q-426 is still unclear, which limits the potential application of Q-426.

**Results:**

Genome sequencing revealed that Q-426 contains a single circular chromosome 4,086,827 bp in length, with 4691 coding sequences and an average GC content of 46.3%. The Q-426 strain has a high degree of collinearity with *B. velezensis* FZB42, *B. velezensis* SQR9, and *B. amyloliquefaciens* DSM7, and the strain was reidentified as *B. velezensis* Q-426 based on the homology analysis results. Many genes in the Q-426 genome have plant growth-promoting activity, including the secondary metabolites of lipopeptides. Genome mining revealed 14 clusters and 732 genes encoding secondary metabolites with predicted functions, including the surfactin, iturin, and fengycin families. In addition, twelve lipopeptides (surfactin, iturin and fengycin) were successfully detected from the fermentation broth of *B. velezensis* Q-426 by ultra-high performance liquid chromatography-quadrupole time-of-flight mass spectrometry (UHPLC–QTOF–MS/MS), which is consistent with the genome analysis results. We found that Q-426 produced indole-3-acetic acid (IAA) at 1.56 mg/l on the third day of incubation, which might promote the growth of plants. Moreover, we identified eighteen volatile compounds (VOCs, including 2-heptanone, 6-methylheptan-2-one, 5-methylheptan-2-one, 2-nonanone, 2-decanone, 2-undecanone, 2-dodecanone, 2-tridecanone, 2-tetradecanone, 2-nonadecanone, pentadecanoic acid, oleic acid, dethyl phthalate, dibutyl phthalate, methyl (9E,12E)-octadeca-9,12-dienoate), pentadecane, (6E,10E)-1,2,3,4,4a,5,8,9,12,12a-decahydro-1,4-methanobenzo[10]annulene, and nonanal) based on gas chromatograph-mass spectrometer (GC/MS) results.

**Conclusions:**

We mined secondary metabolite-related genes from the genome based on whole-genome sequence results. Our study laid the theoretical foundation for the development of secondary metabolites and the application of *B. velezensis* Q-426. Our findings provide insights into the genetic characteristics responsible for the bioactivities and potential application of *B. velezensis* Q-426 as a plant growth-promoting strain in ecological agriculture.

## Background

Recently, the emergence of green and environmentally friendly agriculture has attracted much attention. The common policy requirement is to reduce the intensive use of agrochemical inputs [1], and increase the use proportion of organic fertilizer products and biocontrol agents [2]. Using microorganisms to control plant diseases has been successful in many ways. The strains from the genera *Bacillus*, *Pseudomonas*, *Burkholderia*, *Enterobacter*, *Pasteurella*, and *Agrobacterium* have the most important role in biological control [2, 3]. *Bacillus* species are popular in farm systems due to their typical outstanding advantages, such as storage in warehouse conditions as stable dry powders that have a long shelf life [2, 3]. In addition, *Bacillus* species have the capability of forming endospores that can tolerate heat and drought [2, 3]. As a result, several *Bacillus* strains have been widely used in biological control, including *B. subtilis, B. thuringiensis, B. amyloliquefaciens, B. cereus, B. megaterium, B. licheniformis, B. polymyxa,* and *B. velezensis* [4].

*B. velezensis* is a gram-positive bacterium that is enriched in various environments, including soil, living organisms, intestinal microflora, and deep-sea sediments [5]. Several commercially available strains, such as *B. velezensis* FZB42 [6], *B. velezensis* SQR9 [7], and *B. velezensis* UCMB5113 [8], are currently being used for the efficient control of plant disease with improved productivity of many crops. Chen et al. sequenced the whole genome of *B. velezensis* FZB42 in 2007 [9], and analyzed genes encoding the antimicrobial compounds, responsible for biocontrol effects against plant pathogens [10, 11]. There were 13 gene clusters involved in nonribosomal and ribosomal synthesis of secondary metabolites with putative antimicrobial action in the genome of *B. velezensis* FZB42 [6, 9]. Xu et al. reported that the rhizosphere strain *B. velezensis* SQR9 can produce one of the lipopeptide compounds (bacillomycin D), which plays a crucial role in the antagonistic activity against *Fusarium oxysporum* but also affects the expression of the genes involved in biofilm formation [12]. Chen et al. found that *B. velezensis* SQR9 confers plant salt tolerance by protecting plant cells and managing Na^+^ homeostasis [13]. Hence, *B. velezensis* SQR9 can be used in salt stress-prone areas, thereby promoting agricultural production [13]. Abd El-Daim et al. reported that *B. velezensis* UCMB5113 inoculation resulted in significant metabolic modulation and affected the abundance of several proteins in wheat leaves [8]. The study indicated that *B. velezensis* UCMB5113 utilized similar metabolic and molecular regulatory strategies to enhance the tolerance of wheat exposed to different abiotic stress factors, including heat, cold and drought [8]. These *B. velezensis* strains have been commercialized in the form of fungicides [6–8, 14]. The biocontrol activities were due to the production of secondary metabolites in *B. velezensis*, such as polyketides (including difficidin, bacillaene, and macrolactin) and cyclic lipopeptides (including surfactin, fengycin, bacillibactin, iturin, and bacillomycin), some of which can promote plant growth, control plant pathogens, and induce systemic acquired resistance in plants [14].

*B. velezensis* Q-426 was isolated and purified from compost samples through its antifungal activities in Dalian, China [15]. In a previous study, strain Q-426 was misclassified as *B. amyloliquefaciens* according to morphological and biochemical characteristics and 16S rDNA sequence [15–21]. *B. velezensis* Q-426 was tested for its potential use against a variety of plant pathogens. Zhao et al. screened four genes involved in the biosynthesis of antifungal agents and revealed that the *fen* and *bmy* gene clusters are present in the Q-426 genome [18]. *B. velezensis* Q-426 has been reported to produce diketopiperazines (DKPs) and lipopeptides (such as bacillomycin D, fengycin A, and fengycin B) synthesized by nonribosomal peptide synthetases (NRPS) [15, 18]. Wang et al. found that *B. velezensis* Q-426 produced four kinds of diketopiperazines, which could inhibit biofilm formation at the gas–liquid interface [15]. Moreover, DKPs reduced extracellular polymeric substance (EPS) components, proteins, polysaccharides, DNA, and so on [15]. Zhao et al. studied the correlation between substrates of the medium and lipopeptide production using single-dimensional search techniques [17]. Zhao et al. also evaluated the biosurfactant properties of lipopeptide mixtures using six different methods including bacterial adhesion to hydrocarbon assays, surface tension measurements, lipase activity, oil displacement tests, hemolytic activity and emulsification activity, and found that *B. velezensis* Q-426 may have great potential in agricultural and environmental fields [17]. Tao et al. demonstrated that bacillomycin D is closely related to hemolytic activity and antifungal activity using homologous recombination gene knockout technology [22]. Quan et al. revealed that C-16 bacillomycin D can effectively influence cell migration, and cells treated with C-16 bacillomycin D showed typical apoptotic morphology with increasing drug concentration in early apoptosis, late apoptosis percentage increased, and G_0_/G_1_ arrest was induced significantly [23]. Overall, *B. velezensis* Q-426 is a potential biocontrol agent against fungi and bacteria; however, insufficient knowledge about the whole genome of *B. velezensis* Q-426 has limited its application in agriculture and biotechnology, although some progress has been made in the last decade.

In this study, we aimed to explore the production potential of secondary metabolites and elucidate the biocontrol performance of *B. velezensis* Q-426 based on whole genome information. We performed genome sequencing and mined secondary metabolite related genes and plant growth-promoting activity-related genes from the genome based on the whole genome sequence results. We found that many genes participated in the synthesis of secondary metabolites and that *B. velezensis* Q-426 had great potential in plant growth-promotion. The present study provides a foundation for further studies of related genes and functions and facilitates genetic engineering of *B. velezensis* Q-426 to promote agricultural and industrial applications.

## Results and discussion

### Genomic features of strain Q-426

**Fig. 1:**
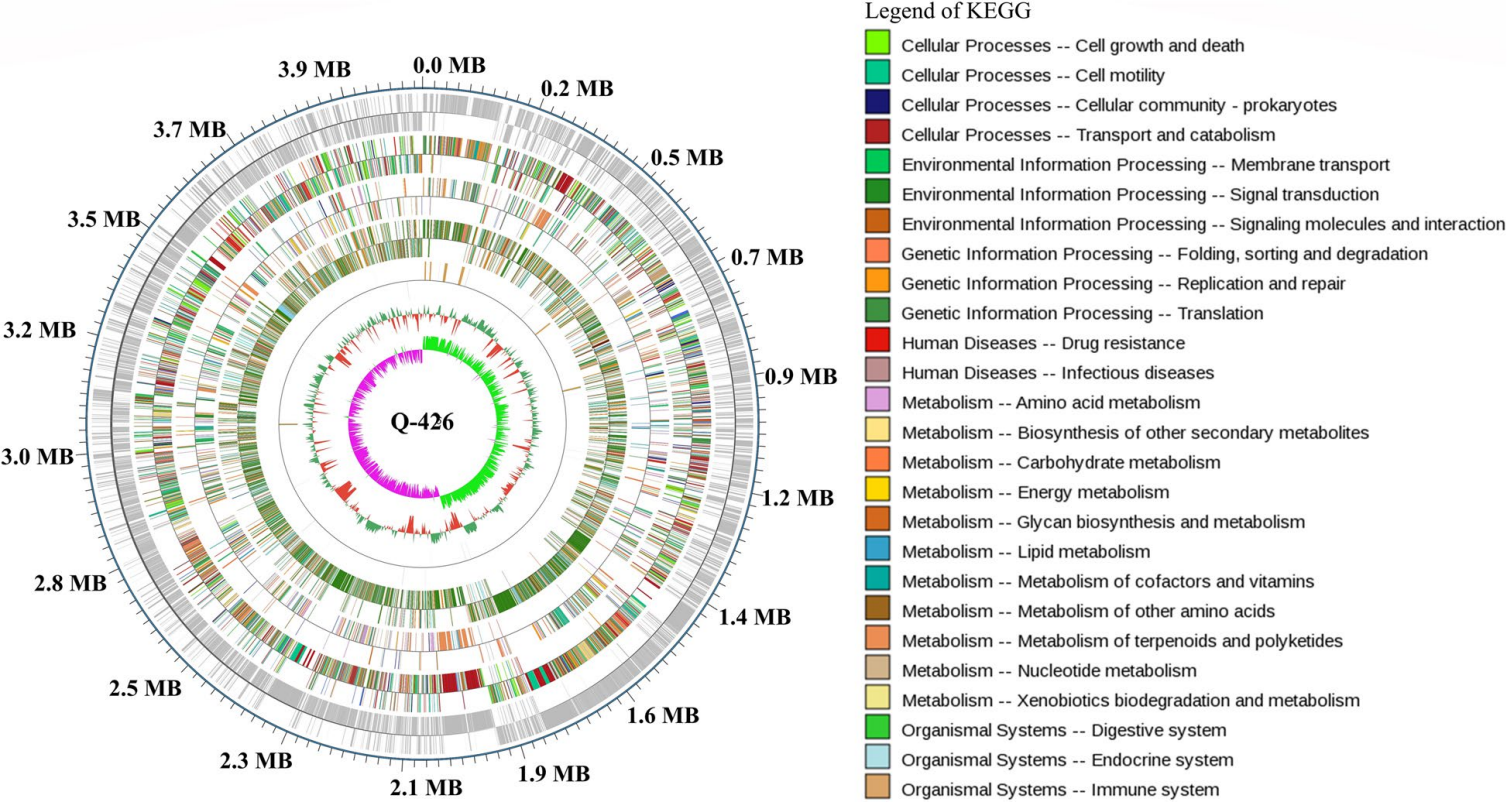
Circular genome maps of strain Q-426 chromosome. From the outer circle to the inner circle, the outermost circle marks the genome size, and the odd and even circles represent the plus- and minus-strand genes, respectively. The first and second circles are coding genes, the third and fourth circles are COG annotation results, the fifth and sixth circles are KEGG annotation results, the seventh and eighth circles are GO annotation results, and the ninth and tenth circles are ncRNA annotation results. The legend of KEGG annotation is shown on the right of the picture

To investigate the genomic and metabolic features of strain Q-426, its whole genome was completely sequenced. Comprehensive genome analysis revealed the presence of 103,490 reads with an average read length of 10,750 bp. One complete circular chromosome (4,086,827 bp) with a contig was assembled, and the average G + C content was 46.3% (Fig. 1). A total of 4691 coding DNA sequences (CDS) genes, 88 tRNA genes, and 27 rRNA genes (16S-23S-5S rRNA) were predicted in this genome. By aligning the genome sequences to six commonly used databases, 4201, 3457, 3041, 3486, 3041, and 4138 of unique genes were matched with sequences in the databases NR, Swiss-Prot, Pfam, COG, GO, and KEGG, respectively.

**Fig. 2:**
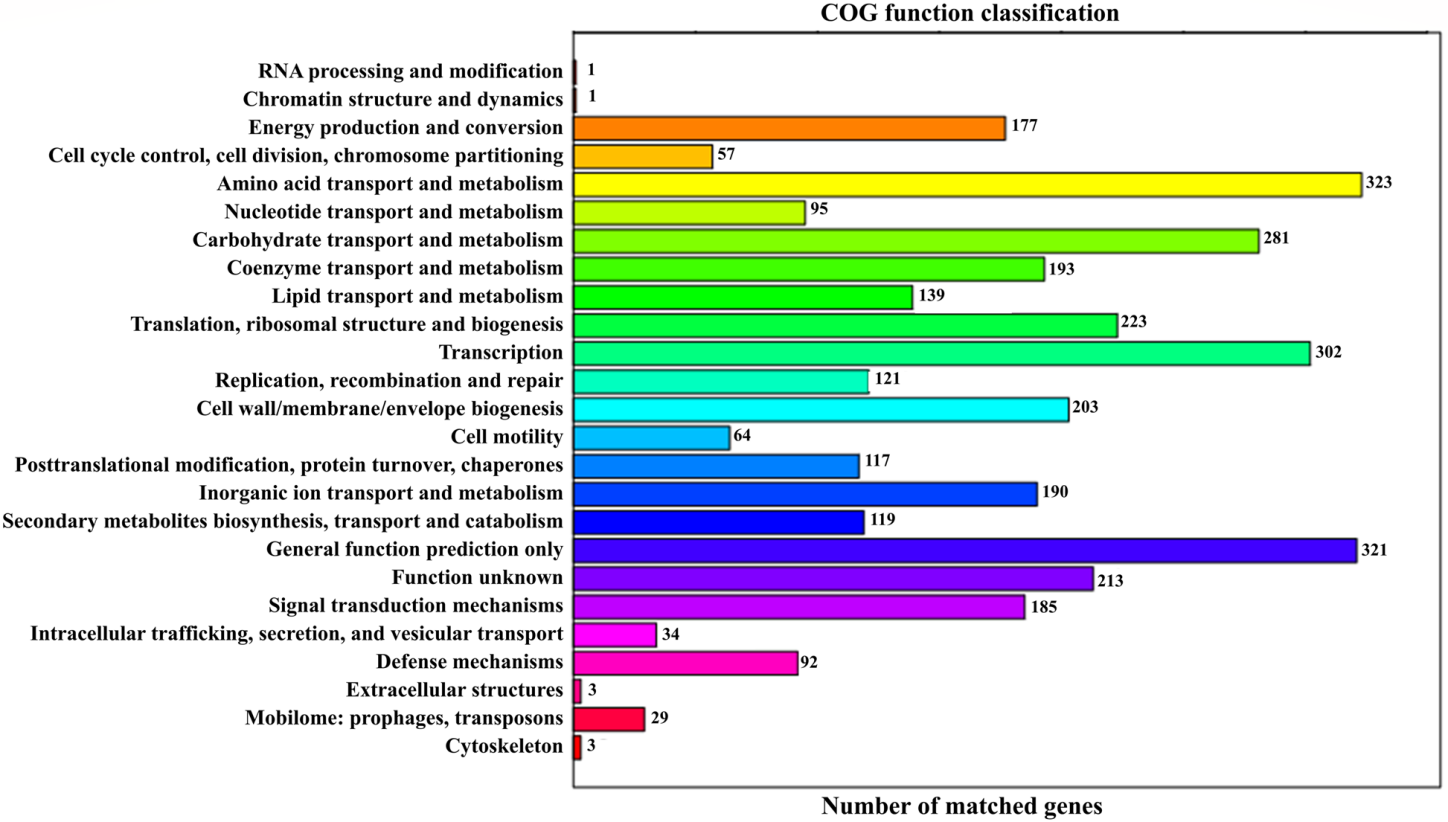
COG annotation of strain Q-426

To further evaluate the completeness of the transcriptome library and the effectiveness of the annotation process, we searched the annotated sequences for the genes involved in COG classifications [24]. The COG analysis shown in Fig. 2 classified the identified genes into 25 categories. The most frequent functional category was amino acid transport and metabolism. The highest number of genes (323) participated in amino acid transport and metabolism, accounting for 9.27% of all annotated protein sequences. A group of 321 genes was involved in general function prediction only, and a group of 302 genes is involved in transcription. These proteins accounted for 9.20% and 8.66% of the total protein sequences, respectively. The annotation results suggested that 119 genes participated in secondary metabolite biosynthesis, transport, and catabolism. As expected, the lipopeptides including surfactin, iturin, and fengycin gene clusters, were found in the genome sequence of Q-426.

**Fig. 3:**
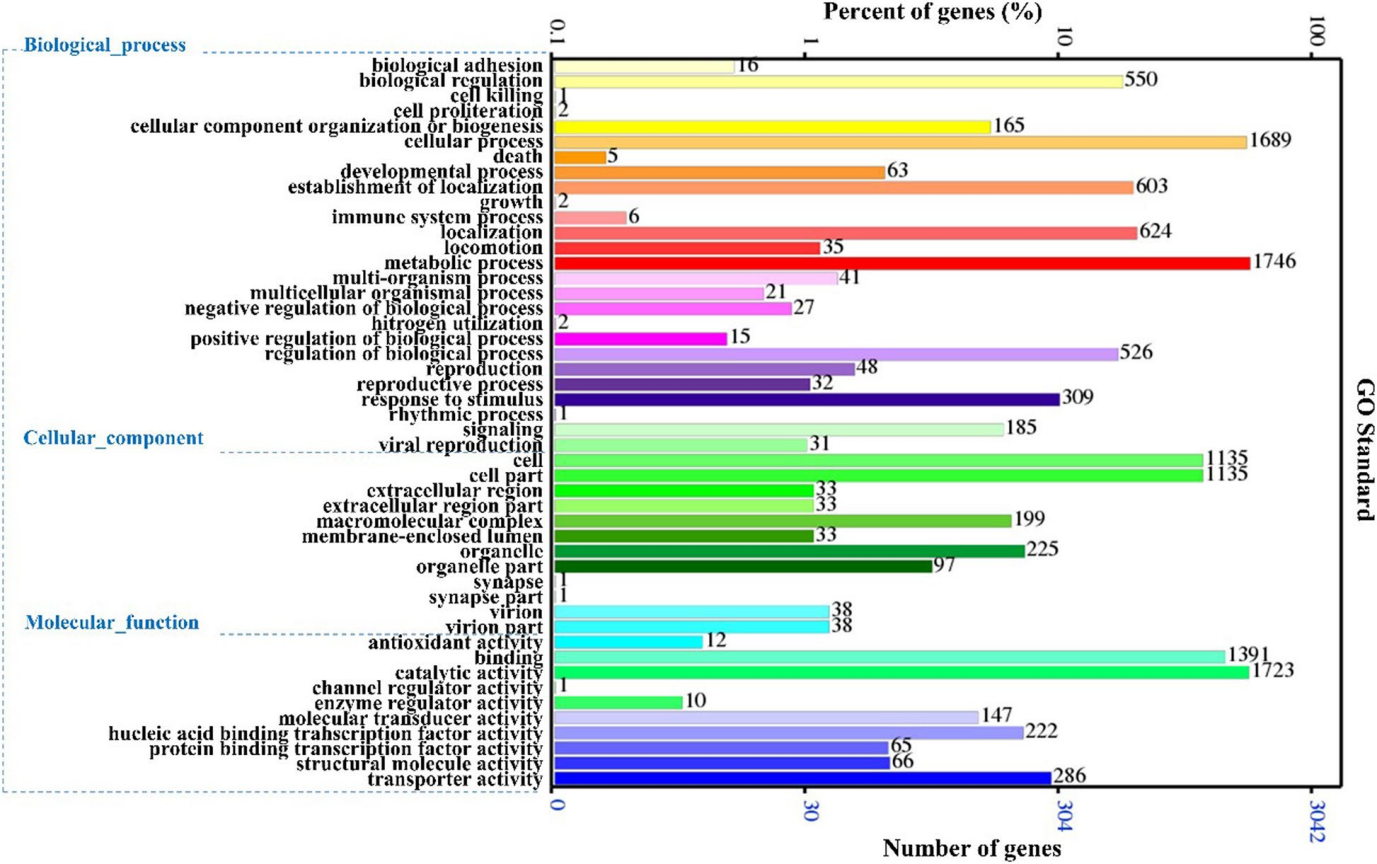
GO annotation of strain Q-426

In the present study, we used the InterProScan database [25] to predict all of the protein domains and functional sites of *B. velezensis* Q-426 and extract the information of GO [26, 27]. Based on the GO functional annotation shown in Fig. 3, the identified proteins were classified into 3 large groups, including biological process, cellular components, and molecular functions. The results suggested that the proteins were annotated into 48 functional groups, including 26 biological processes, 12 cellular components, and 10 molecular functions (Fig. 3). In biological processes, the proteins were mainly involved in metabolic processes (1746 genes), cellular processes (1689 genes), and localization (624 genes). In cellular components, the proteins were mainly involved in cell (1135 genes), cell part (1135 genes), and organelle (225 genes). In molecular functions, the proteins were mainly involved in catalytic activity (1723 genes), binding (1391 genes), and transporter activity (286 genes).

**Fig. 4:**
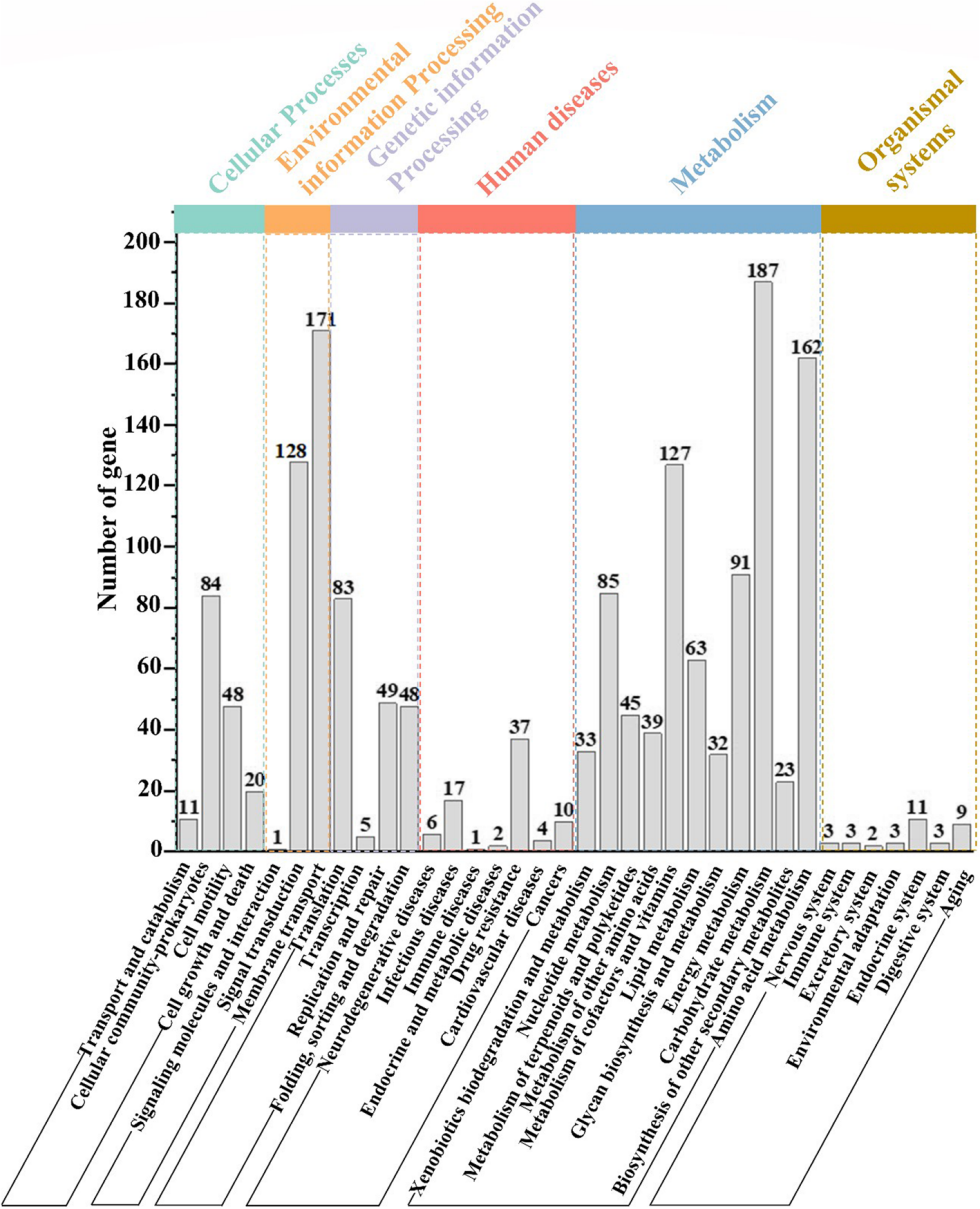
KEGG annotation of strain Q-426. There are six categories shown in the upper of the figure, and each category is divided into a secondary classification system. The X-axis is the biological pathway, and the Y-axis is the number of genes. For secondary classification, different colors are used to distinguish the primary classification of biological pathways

The genes involved in the metabolic pathways were analyzed statistically using the KEGG analysis tool in the genome sequence of *B. velezensis* Q-426 [28]. The KEGG analysis results showed that the proteins were annotated to 36 KEGG pathways, classified into 6 large groups, including cellular processes (163 genes), environmental information processing (300 genes), genetic information processing (185 genes), human diseases (77 genes), metabolism (887 genes), and organismal systems (34 genes) (Fig. 4). Carbohydrate metabolism pathways (187 genes) and membrane transport (171 genes) were the primary enriched pathways, followed by amino acid metabolism (162 genes). The KEGG database analysis showed a great number of two-component systems (114 genes) and ABC transporters (135 genes). Moreover, 76 genes were related to quorum sensing systems, which played a crucial role in sporulation, biofilm formation, and genetic competence [29, 30].

### The taxonomic status of strain Q-426

**Fig. 5:**
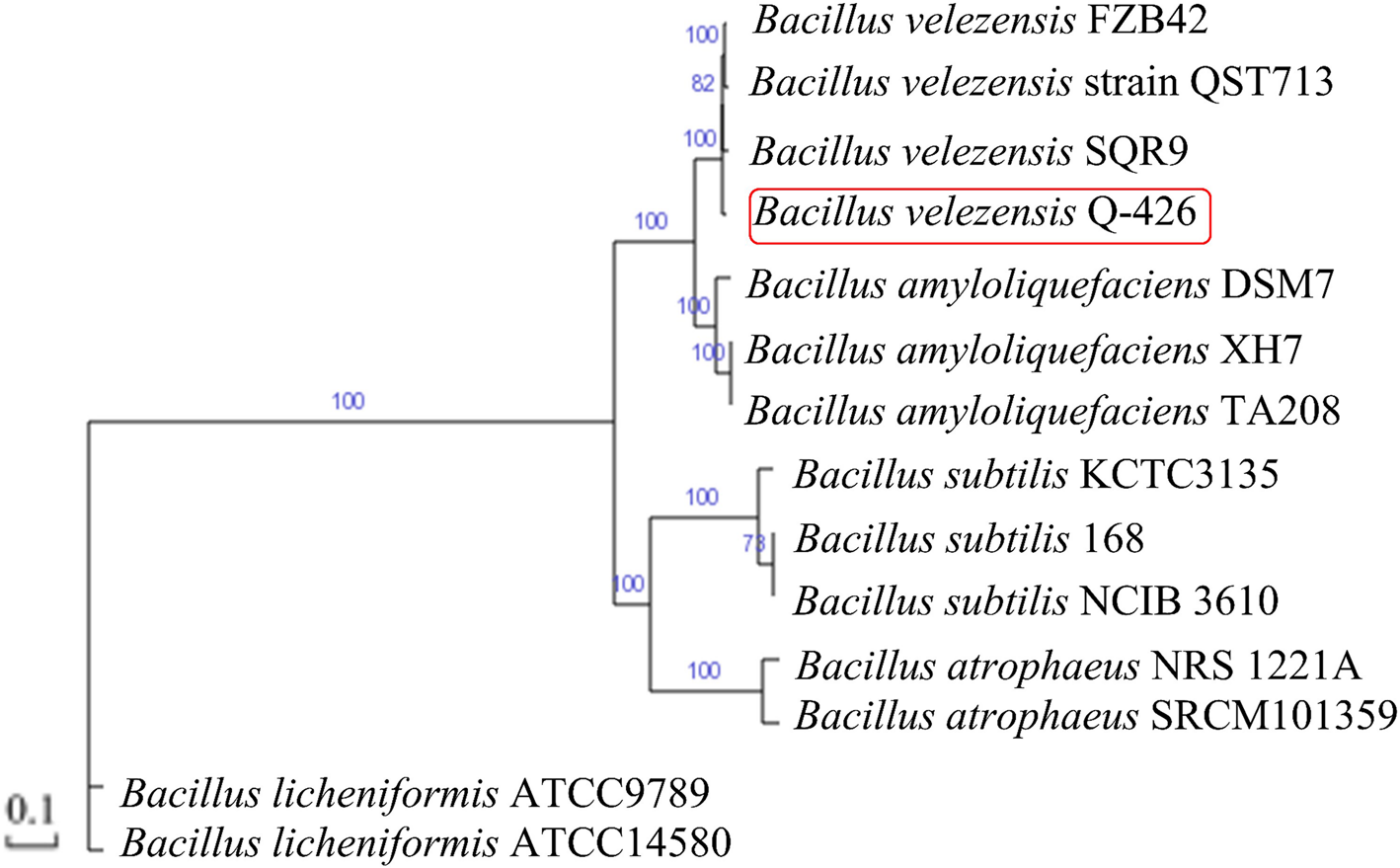
Phylogenetic tree of the Q-426 strain based on whole-genome alignments

The phylogenetic analysis of strain Q-426 based on whole genomic sequences was conducted with related *Bacillus* species (Fig. 5) to show the phylogenetic relationships. In previous study, strain Q-426 was identified as *B. amyloliquefaciens* based on 16S rDNA sequences. However, based on the comparative phylogenomic analysis of *Bacillus* genomes strain Q-426 should be reassigned as *B. velezensis* species. The phylogenetic tree indicated that Q-426 was evolutionarily closest to *B. velezensis* SQR9. *B. velezensis* FZB42 and *B. velezensis* QST713 were clustered together. Therefore, Q-426 was reclassified as *B. velezensis* Q-426.

*B. velezensis* and *B. amyloliquefaciens* have similar morphological, biochemical, physiological, phenotypic, and phylogenetic characteristics [31]. They are closely related members of the “operational Group *B. amyloliquefaciens*”, a taxonomical unit above the species level within the “*B. subtilis* species complex” [31]. As a result, it was quite difficult to separate these two taxa from each other. For example, the plant growth-promoting model bacterium *B. velezensis* FZB42 was regarded as the type strain of *B. amyloliquefaciens* [32], and it was recognized as *B. velezensis* FZB42 based on phylogenomic analysis five years later [33]. Another typical example is *B. velezensis* DSM7, which was first identified as *B. amyloliquefaciens* [34] and then regarded as *B. amyloliquefaciens* subsp. *plantarum* three years later [32]. Finally, it was reclassified five years later based on DNA-DNA hybridization, as well as phenotypic and phylogenetic analyses [31, 35]. With the development of complete genome sequencing technology, it has been widely approved to verify taxonomic status based on whole-genome alignments results.

### Comparison of genetic characteristics between *B. velezensis *Q-426 and *four other reference Bacillus strains*

*Bacillus* spp. is one of the most extensively studied beneficial microorganisms in the rhizosphere, and several commercial products belonging to *Bacillus* are currently available for agricultural production [36]. Researchers have studied the antimicrobial compounds in *B. velezensis* FZB42, that are responsible for biocontrol effects against plant pathogens [10, 11]. The study of *B. velezensis* SQR9 suggested that it has the ability to promote agricultural production [13]. We further analyzed the genetic characteristics of *B. velezensis* Q-426 and four other highly active *Bacillus* strains, including *B. velezensis* FZB42 [6], *B. velezensis* SQR9 [7], *B. amyloliquefaciens* type strain DSM7 [37], and type strain *B. subtilis* 168 [38]. The general features of these strains are illustrated in Table 1. A comparative analysis among the GC contents suggested that Q-426 shared similar values with *B. velezensis* FZB42 [6], *B. velezensis* SQR9 [7], and *B. amyloliquefaciens* DSM7 [37], which was much higher than that of *B. subtilis* 168 [38]. The CDS number of Q-426 was 4691 and was larger than four reference *Bacillus* strains. Secondary metabolite analysis with antiSMASH predicted 14 gene clusters related to secondary metabolism in Q-426, and the cluster numbers in *B. velezensis* FZB42 [6], *B. velezensis* SQR9 [7], *B. amyloliquefaciens* DSM7 [37], and *B. subtilis* 168 [38] were 13, 12, 11, and 6, respectively. The results suggested that the gene encoding secondary metabolite synthesis in *B. velezensis* and *B. amyloliquefaciens* was much more abundant than that in *B. subtilis*.

**Table 1:** Comparison of genomic features between Q-426 and other *Bacillus* strains

Strain name	Q-426	FZB42 [6]	SQR9 [7]	DSM7 [37]	168 [38]
Genome size	4,086,827	3,918,596	4,117,023	3,980,199	4,215,606
GC content (%)	46.30	46.40	46.10	46.10	43.50
CDS number	4691	3693	4078	3921	4106
Average CDS length	782	933	916	888	895
tRNA number	86	89	72	94	86
Secondary metabolite synthesis gene clusters					
Gene length	316,610	771,823	811,617	559,717	144,451
Cluster number	14	13	12	11	6
Proportion of total length (%)	7.75	19.70	19.71	14.06	3.43

**Fig. 6:**
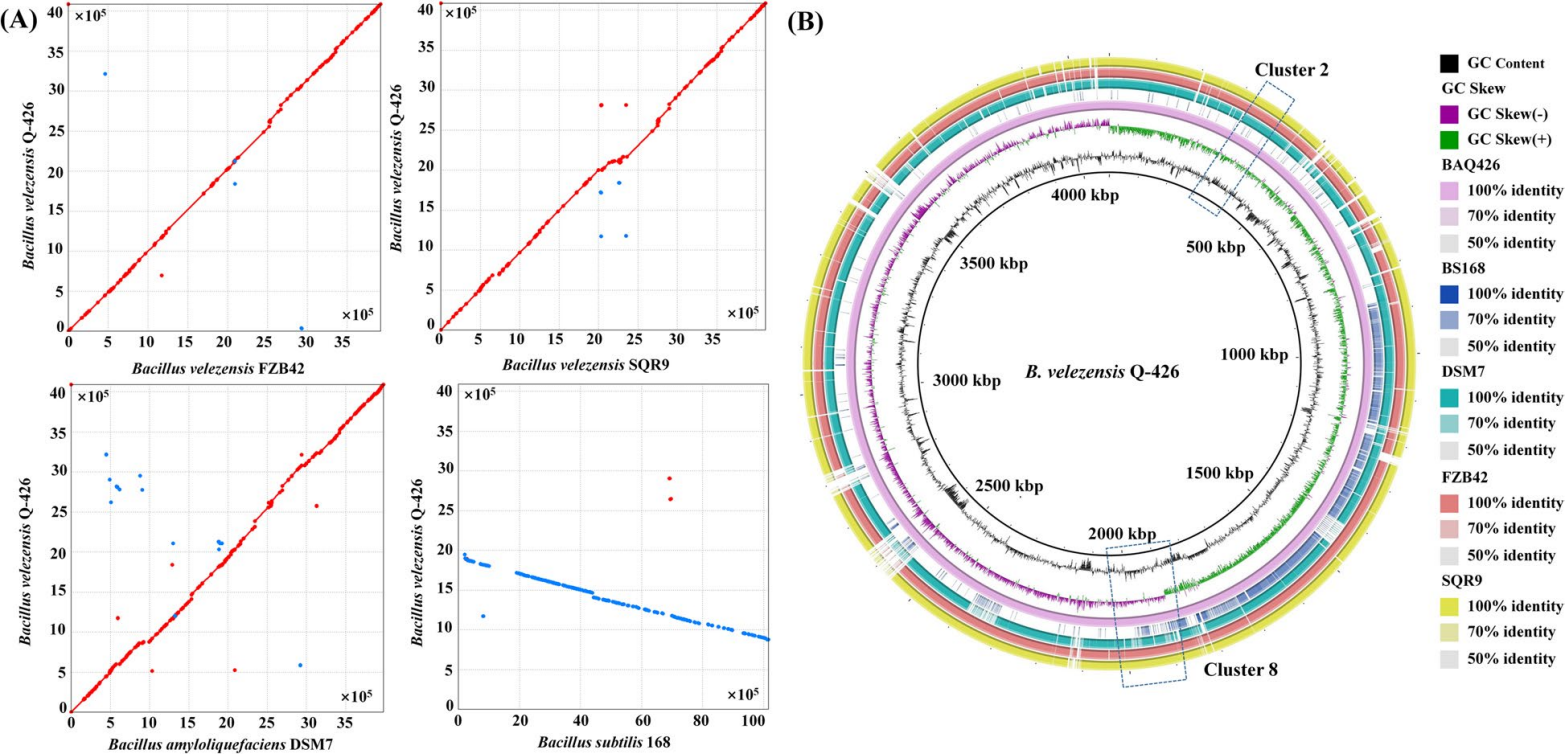
Genome comparison of *B. velezensis* Q-426 and its homologous strains. **A** Collinearity analysis. **B** Circular genome blast atlas. From the outer circle to the inner circle, the outermost circle marks the whole genome of *B. velezensis* SQR9, the second circle marks the whole genome of *B. velezensis* FZB42, the third circle marks the whole genome of *B. amyloliquefaciens* DSM7, and the fourth circle marks the whole genome of *B. subtilis* 168. They were aligned with *B. velezensis* Q-426 (inner circle) using BRIG

In comparative genomics, synteny and collinearity analysis are two effective methods to evaluate the colocalization of genetic loci among various species. Synteny and collinearity analysis is recognized as a standard step for comparative genomics research [39, 40]. To understand the relatedness of Q-426 to other *Bacillus* strains, we performed whole-genome collinearity analysis among *B. velezensis* Q-426 and four other kinds of highly active *Bacillus* strains, such as *B. velezensis* FZB42 [6], *B. velezensis* SQR9 [7], *B. amyloliquefaciens* DSM7 [37], and *B. subtilis* 168 [38]. The genomes of *B. velezensis* Q-426 presented high collinearity with the *B. velezensis* FZB42, *B. velezensis* SQR9, and *B. amyloliquefaciens* DSM7 genomes but did not show collinearity with *B. subtilis* 168 (Fig. 6A). To reveal the differences at the nucleotide level, the genome of Q-426 was used as a reference to align the fully sequenced genomes of the other four *Bacillus* strains using BRIG. The results also revealed that the Q-426 genome sequence has high similarity to the genome sequences of *B. velezensis* FZB42, *B. velezensis* SQR9, and *B. amyloliquefaciens* DSM7 (Fig. 6B).

### Secondary metabolite biosynthetic gene clusters in strain Q-426

**Fig. 7:**
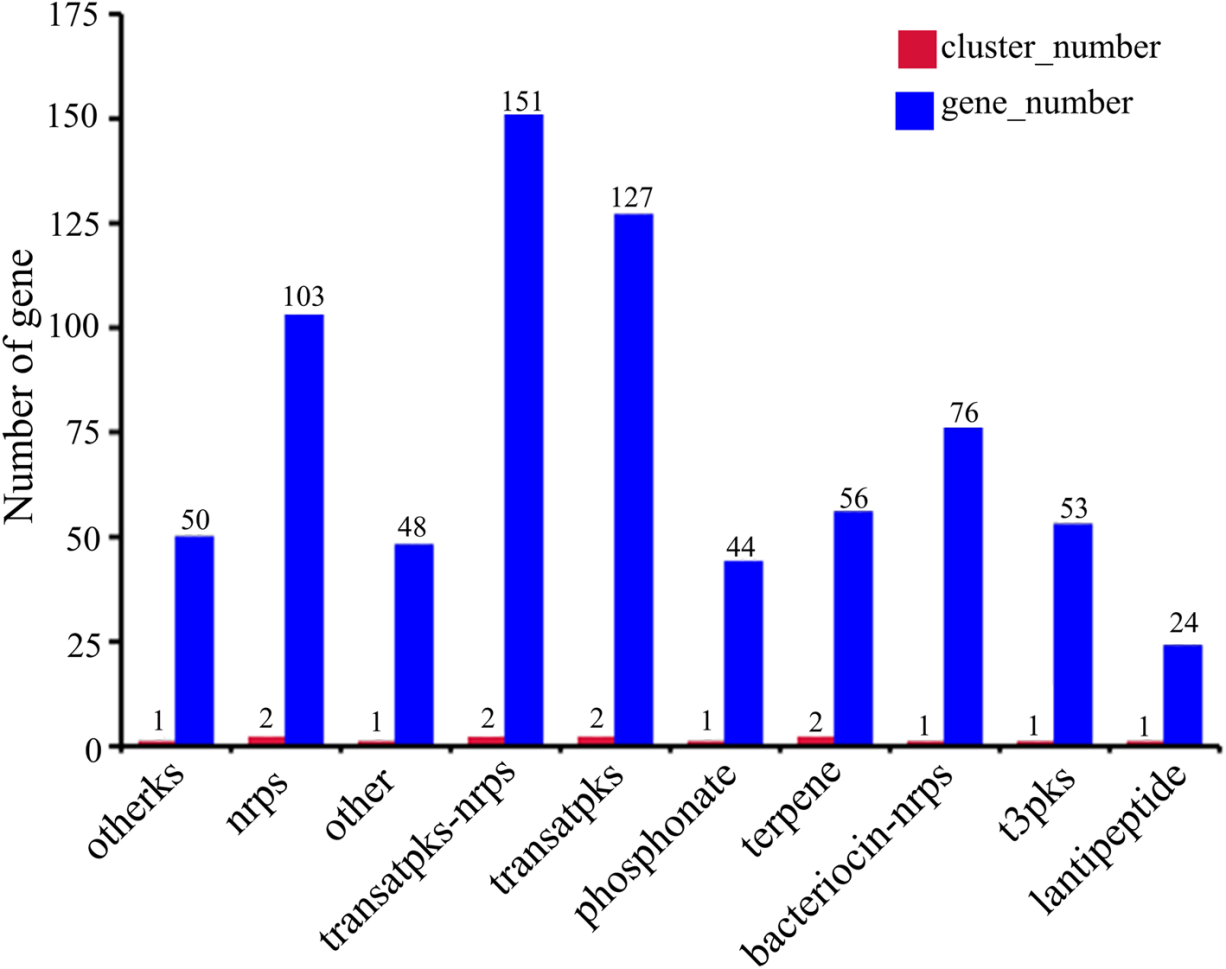
Quantitative analysis of secondary metabolite genes. Otherks: other ketides; nrps: nonribosomal peptide synthetase; transatpks: trans-acyl transferase polyketide synthetases; pks: polyketide synthase; t3pks: type III polyketide

It was reported that *B. velezensis* has an impressive capacity to produce secondary metabolites with antimicrobial activities, including lipopeptides (surfactin, fengycin, and bacillomycin D), polyketides (macrolactin, bacillaene, and difficidin or oxydifficidin), and peptides (plantazolicin, amylocyclicin, and bacilysin). With advances in sequencing technologies and the development of robust genome mining tools, people have more options for research on secondary metabolites. The antibiotics and secondary metabolite analysis shell (antiSMASH) can quickly annotate and analyze secondary metabolite biosynthetic gene clusters, and helps to estimate the types of compounds encoded by the gene clusters [14, 41]. The secondary metabolite biosynthetic gene clusters in the genome of *B. velezensis* Q-426 were predicted using antiSMASH 4.0. In total, the antiSMASH analysis identified 14 clusters of secondary metabolites, including two nrps clusters, two terpene clusters, two transatpks clusters, two transatpks-nrps clusters, one lantipeptide cluster, one phosphonate cluster, one otherks cluster, one t3pks cluster, one bacteriocin-nrps cluster, and one gene cluster with unknown function named the other cluster (Fig. 7).

**Fig. 8:**
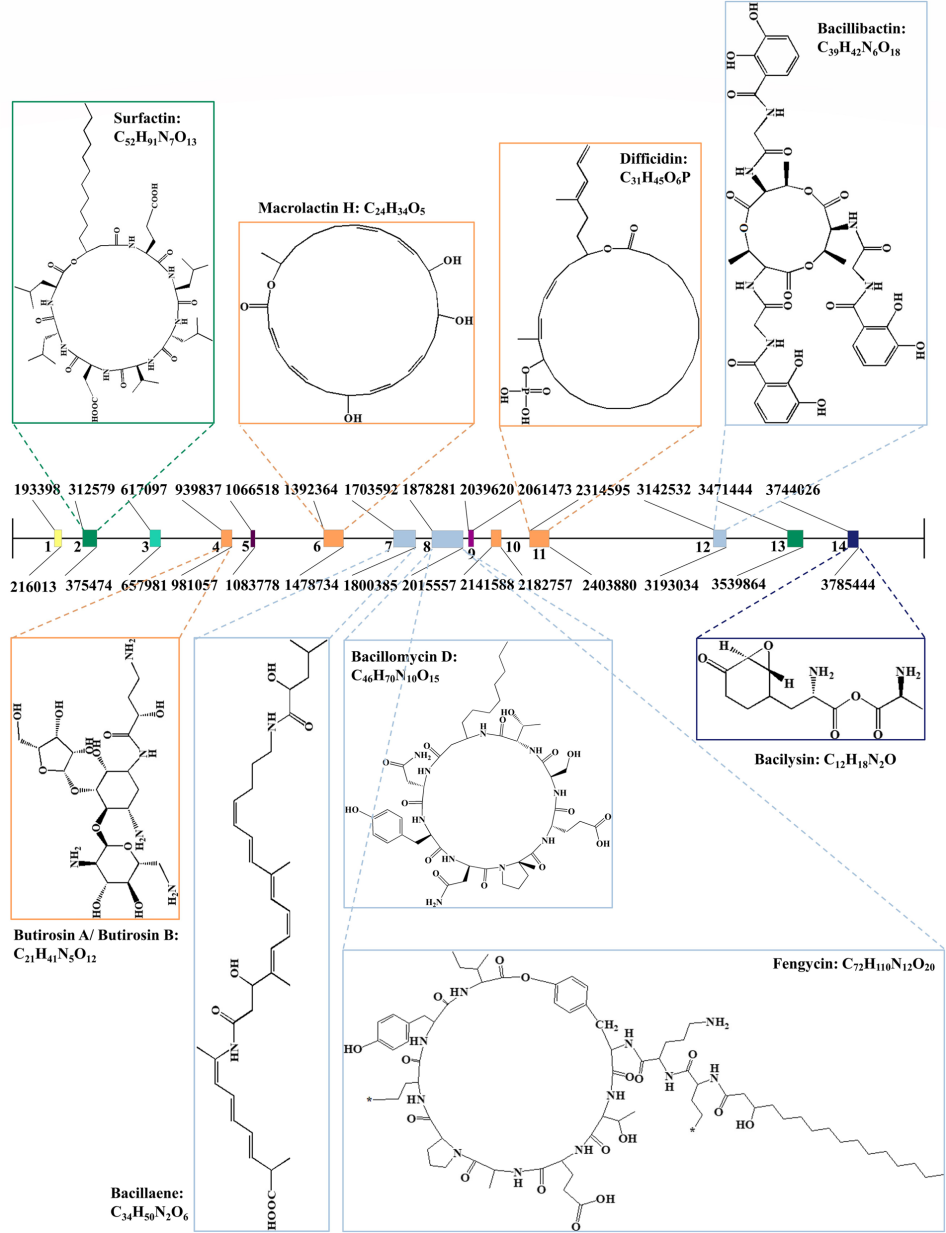
Secondary metabolite gene clusters identified in *B. velezensis* Q-426 genome using antiSMASH software version 4.0

Among them, five clusters had 100% amino acid sequence homology with known gene clusters, including transatpks (Cluster 6), transatpks-nrps (Clusters 7 and 8), bacteriocin-nrps (Cluster 12), and others (Cluster 14). The above five clusters could synthesize macrolactin H, bacillaene, fengycin, bacillibactin, and bacilysin, respectively (Table 2, Fig. 7). The gene in nrps (Cluster 2) showed 82% amino acid similarity with a surfactin synthetase gene cluster. The gene in transatpks (Cluster 11) showed 93% amino acid similarity with the difficidin synthetase gene cluster. However, phosphonate (Cluster 3), terpene (Clusters 5 and 9), t3pks (Cluster 10), and nrps (Cluster13) did not match any known gene clusters (Table 2, Fig. 7). Comparison of gene clusters among *B. velezensis* Q-426 and the other four *Bacillus* strains (FZB42 [6], SQR9 [7], DSM7 [37], and 168 [38]) illustrated that there are five highly conserved clusters (Clusters 2, 5, 9, 10, and 13) (Table 2). Ten clusters (Clusters 2, 5–11, 13 and 14) are conserved by the four strains of *B. velezensis* Q-426, *B. velezensis* FZB42, *B. velezensis* SQR9, and *B. amyloliquefaciens* DSM7 (Table 2). Two clusters (lantipeptide and phosphonate) are not conserved by the three strains of *B. velezensis* (Q-426, FZB42, and SQR9) (Table 2). Through genome mining, clusters responsible for the synthesis of secondary metabolites were identified in the genome of *B. velezensis* Q-426, and the clusters responsible for the synthesis of surfactin, butirosin A/butirosin B, macrolactin H, bacillaene, fengycin, bacillomycin D, difficidin, bacillibactin, and bacilysin were detected. The structures of these secondary metabolites are also shown in Fig. 8.

**Table 2:** Genetic clusters coding for secondary metabolites in the genome of Q-426

Q-426		Gene cluster location				Presence ( +) or absence (-)				
Cluster number	Cluster name	From	To	Most similar known cluster	Similarity (%)	Bv FZB42 [6]	Bv SQR9 [7]	Ba DSM7 [37]	Bs 168 [38]	
1	lantipeptide	193398	216013	locillomycin	35	**-**	**-**	** + **		**-**
2	nrps	312579	375474	surfactin	82	** + **	** + **	** + **		**+ **
3	phosphonate	617097	657981	unknown	**-**	** + **	**-**	**-**		**-**
4	otherks	939837	981057	butirosin A/ B	7	** + **	** + **	**-**		**-**
5	terpene	1066518	1083778	unknown	**-**	** + **	** + **	** + **		**+ **
6	transatpks	1392364	1478734	macrolactin H	100	** + **	** + **	** + **		**-**
7	transatpks-nrps	1703592	1800385	bacillaene	100	** + **	** + **	** + **		**-**
8	transatpks-nrps	1878281	2015557	fengycin, iturin	100	** + **	** + **	** + **		**-**
9	terpene	2039620	2141588	unknown	**-**	** + **	** + **	** + **		**+ **
10	t3pks	2061473	2182757	unknown	**-**	** + **	** + **	** + **		**+ **
11	transatpks	2314595	2403880	difficidin	93	** + **	** + **	** + **		**-**
12	bacteriocin-nrps	3142532	3193034	bacillibactin	100	** + **	** + **	**-**		**-**
13	nrps	3471444	3539864	unknown	**-**	** + **	** + **	** + **		**+ **
14	other	3744026	3785444	bacilysin	100	** + **	** + **	** + **		**+ **

**Fig. 9:**
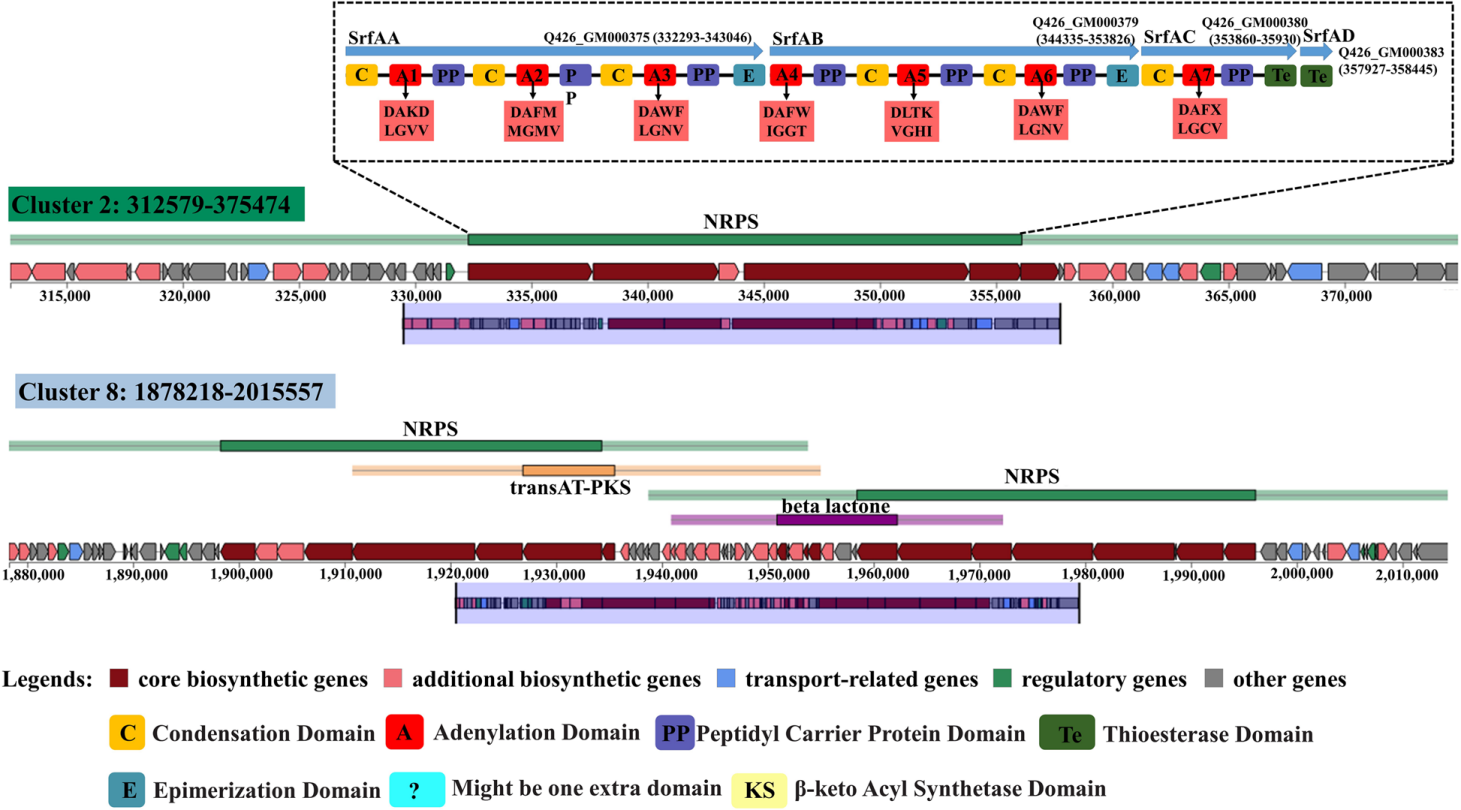
Schematic diagram of gene Clusters 2 and 8 in *B. velezensis* Q-426. The potential secondary metabolite biosynthetic gene clusters were predicted using antiSMASH. Genes with different functions are marked in different color blocks, which are shown in the legend. The functional domains of surfactin synthesis-related genes were analyzed using PKS/NRPS analysis. The abbreviations indicate the functions of the corresponding structural domains. The conserved binding pockets for substrates formed by amino acids in different adenylation domains are shown in light red boxes

The structural compositions of gene Clusters 2 and 8 are shown in Fig. 8, and they were composed of genes for the lipopeptide synthesis, such as surfactin, iturin, and fengycin. The surfactin biosynthetic gene Clusters Q426_GM000375, Q426_GM000379, Q426_GM000380, and Q426_GM000383 in *B. velezensis* Q-426 were analyzed using PRISM, and the core genes were selected for PKS/NRPS analysis (Fig. 9). The surfactin biosynthetic gene cluster contained four genes, including *SurfAA*, *SurfAB*, *SurfAC*, and *SurfAD*. The iturin biosynthetic gene cluster contained three genes of *ituA* (Q426_GM002145), *ituB* (Q426_GM002143), and *ituC* (Q426_GM002139). The fengycin biosynthetic gene cluster contained five genes, *fenE* (Q426_GM002176), *fenD* (Q426_GM002177 and Q426_GM002178), *fenC* (Q426_GM002179), *fenB* (Q426_GM002180), and *fenA* (Q426_GM002182).

Seven kinds of secondary metabolites belonging to iturin (C14-, C15-, and C16-bacillomycin D) and fengycin (C15-, C16-, C17-fengycin A and C17-fengycin B) have been separated and identified in our previous studies, but the surfactin was not found in the fermentation broth [18, 21]. However, we found that a surfactin synthesis gene cluster was contained in the genome of *B. velezensis* Q-426, so the gene cluster was analyzed in detail. The surfactin biosynthetic gene cluster in strain Q-426 contained seven adenylation domains, which were named A1-A7. The crucial amino acid sequences corresponding to the A1-A7 substrate binding cavity were DAKDLGVV, DAFMMGMV, DAWFLGNV, DAFWIGGT, DLTKVGHI, DAWFLGNV, and DAFXLGCV (Fig. 9). Moreover, the potential substrates of A1-A7 were Glu/Asp, Leu/Ile/Val, Leu, Val, Asp, Glu/Asp, and Leu/Ile/Val, respectively (Fig. 9). Collectively, the amino acid sequence of the surfactin generated by Q-426 could be Glu/Asp-Leu/Ile/Val-Leu-Val-Asp-Glu/Asp-Leu/Ile/Val.

### MS/MS analysis of lipopeptides in Q-426

**Fig. 10:**
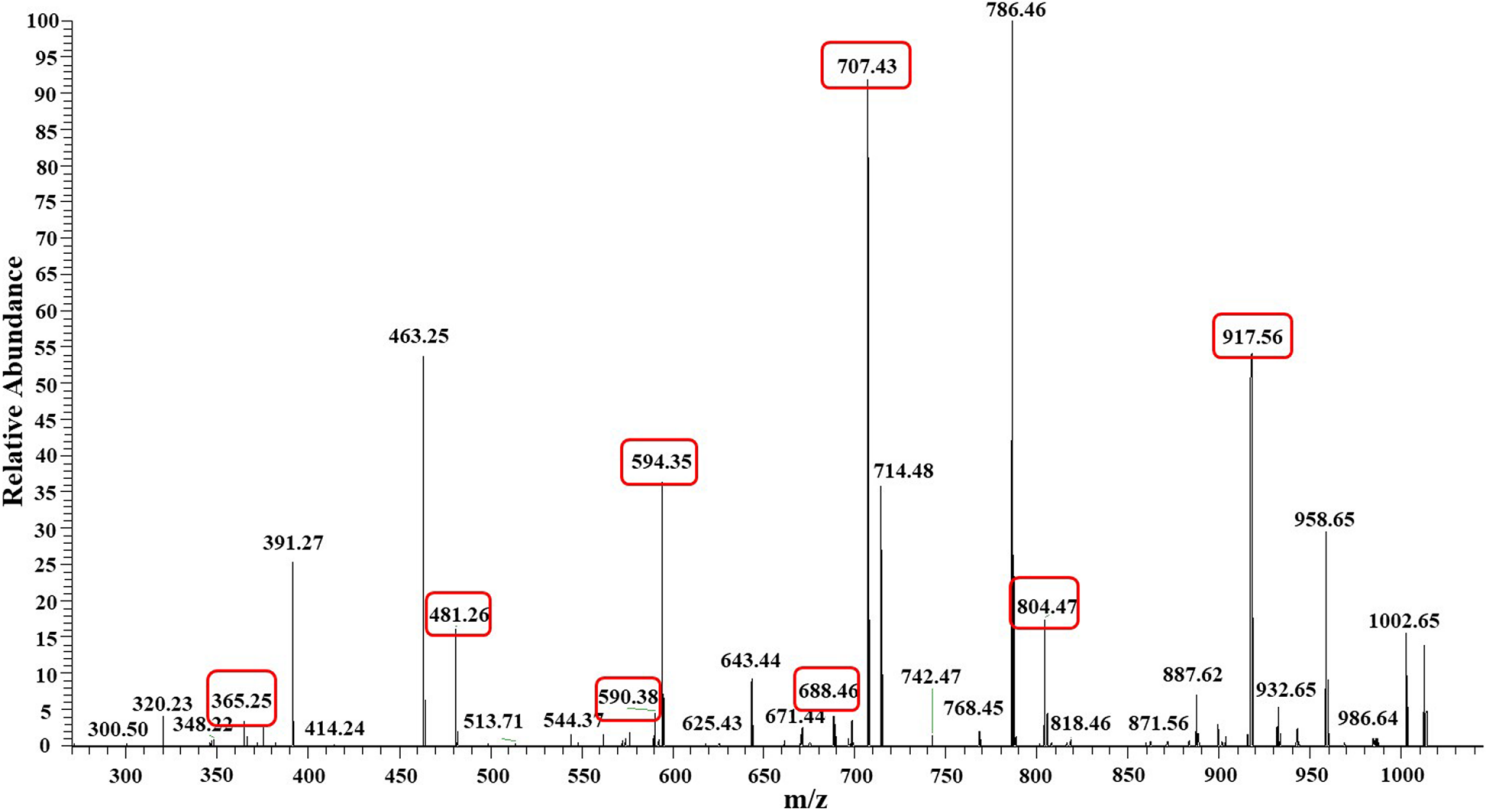
Secondary mass spectrum of precursor ion of surfactin with precursor ion [M + H].^+^ at m/z 1030.64

The structures of lipopeptides from the fermentation broth of *B. velezensis* Q-426 in bran medium were determined by high‒resolution LC‒MS/MS in negative-ion mode. The precursor ions [M + Na]^+^ at m/z 1030.6410, 1030.6410, 1044.6569, 1044.6575, 1058.6715, 1058.6716, 1463.8041, 1491.8368, 1477.8233, 1017.5313, 1031.6415, and 1045.5660 indicated the existence of three types of lipopeptides, including surfactins, iturins and fengycins. The identification of these compounds was mainly according to the typical product ions in the MS/MS of the precursor ions in each compound described by previous studies [42, 43]. For example, the Compund 1 which eluted at 38.33 min in HPLC showed precursor ion m/z [M + Na]^+^ at 1030.6410. As shown in Fig. 10, The ions at m/z 590.38 Da, 689.46 Da, 804.47 Da, and 917.56 Da are generated by β-hydroxy fatty acid Glu-Leu-Leu, β-hydroxy fatty acid-Glu-Leu-Leu-Val, β-hydroxy fatty acid-Glu-Leu-Leu-Val-Asp, and β-hydroxy fatty acid-Glu-Leu-Leu-Val-Asp-Leu, respectively. Ions at m/z 383.25 Da, 481.26 Da, 594.35 Da, and 707.43 Da found in the spectrum are generated by Asp-Leu-Leu, Val-Asp-Leu-Leu, Leu-Val-Asp-Leu-Leu, and Leu-Leu-Val-Asp-Leu-Leu. Collectively, the existence of these characteristic peaks indicates that the structure of 1 was β-C13-Glu-Leu-Leu-Val-Asp-Leu- Ile/Ile, and it was identified as C13-surfactin. Compound 2, whose retention time was different from that of 1, showed the same precursor ion as Compound 1, and the production ions were almost identical. As, Leu, the amino acid at positions 2, 3 and 7 of surfactin, may be replaced by its isomer Ile, which cannot be distinguished by the MS data alone. Thus, Compound 2 was identified as the isomer of C13-surfactin. Accordingly, the other 10 compounds were also indentified as summarized at Table 3. Thus, there were 12 kinds of lipopeptides produced by *B. velezensis* Q-426, including six surfactins (two kinds of C13-surfactin, two kinds of C14-surfactin, and two kinds of C15-surfactin), three iturin (one C13-bacillomycin D, one C14-bacillomycinD, and one C15-bacillomycin D), and three fengycin (one C16-fengycin A, one C16-fengycin B, and one C17-fengycin A).

**Table 3:** The identification of secondary metabolites in Q-426

No.	name	RT/min	m/z	ppm	typical product ions
1	C13-Surfactin	38.33	1030.6410	1.0222	917.5586, 804.4060, 590.4240, 707.4305, 594.3467, 481.2188, 365.2400, 688.5582
2		38.74	1030.6410	1.0222	917.5568, 804.4728, 590.3780, 707.4305, 594.3459, 481.2636, 365.2516, 688.4607
3	C14-Surfactin	40.09	1044.6569	1.0219	931.5721, 594.3448, 972.6702, 728.4932
4		40.85	1044.6575	1.0219	931.5732, 972.6725 728.4938
5	C15-Surfactin	41.89	1058.6715	1.0216	945.5861, 814.4935, 707.4300, 594.3465, 986.6858, 742.5082
6		43.00	1058.6716	1.0216	945.5869, 814.4932, 707.4302, 594.3463, 986.6863, 743.5118
7	C16-Fengycin A	20.17	1463.8041	1.0001	996.4537, 1080.5342, 1144.6583,
8	C16-Fengycin B	20.31	1491.8368	1.0000	994.4878, 1108.5673
9	C17-Fengycin A	21.05	1477.8233	1.0001	966.4025, 1108.5300
10	C13-Bacillomycin D	14.81	1017.5313	0.9965	603.35, 625.3914, 704.1019, 740.4687, 791.4575, 903.2043
11	C14-Bacillomycin D	16.67	1031.6415	0.9967	617.0829, 754.4219, 917.5571
12	C15-Bacillomycin D	17.96	1045.5660	0.9994	631. 4584, 768.6399

In a previous study, *B. velezensis* Q-426 was isolated, and seven kinds of cyclic lipopeptides were identified from the fermentation broth of *B. velezensis* Q-426 cultured in Luria–Bertani, including three iturin (C14-bacillomycin D, C15-bacillomycin D, and C16-bacillomycin D) and four fengycin (C15-fengycin A, C16-fengycin A, C17-fengycin A, and C17-fengycin B) [16, 17]. In this study, we changed the medium (from LB to bran medium) and fermentation conditions (from 37 ℃ in liquid cultures to 28 ℃ in solid cultures). The results suggested that there were three kinds of cyclic lipopeptides produced by *B. velezensis* Q-426 in bran medium, including six surfactin, three iturin, and three fengycin. The composition of the medium and the fermentation conditions might affect the amount and kinds of secondary metabolites.

### Genetic basis for the plant growth-promoting activity of Q-426

Researchers have proposed the term plant growth-promoting rhizobacteria (PGPR) to refer bacteria that inhabit the rhizosphere of plants, and are able to promote plant growth significantly [44]. PGPR promote plant growth through various mechanisms, and it has been proposed that PGPR can synthesize and release plant-promoting compounds, volatile organic compounds, and lipopetide-type compounds and induce systemic resistance [44, 45]. There are several plant-promoting compounds, such as indole-3-acetic acid (IAA), tryptophan, siderophores, cellulase, protease, amylase, β-1,3-glucanase, and tryptophan [14]. Genome analysis revealed the presence of gene clusters for the biosynthesis of plant-promotion and antifungal compounds, such as IAA, tryptophan, siderophores, and phenazine. The Q-426 genome has multiple genes encoding proteins predicted to be associated with plant growth-promoting activity, which are illustrated in Table 4. There are 7 putative genes involved in the production of indole-3-acetic acid (IAA). There are also other genes encoding proteins involved in the production of the volatile compound (VOC) 3-hydroxy-2-butanone, such as acetolactate decarboxylase (*alsD*), alpha-acetolactate synthase (*ilvB*), acetoin dehydrogenase (*acuC*), and 2,3-butanediol dehydrogenase (*bdhA*). The Q-426 genome possesses 3 genes involved in biofilm formation, development, and regulation, including the cell fate regulator YmcA (*ymcA*), scyllo-inositol 2-dehydrogenase NADP ( +) (*iolU*), and regulator of the extracellular matrix (*slrR*). Furthermore, 13 putative genes encoded proteins involved in the synthesis of lipopeptides, such as surfactin, iturin, and fengycin.

**Table 4:** Genes involved in plant growth-promoting activity in Q-426

Gene ID	Gene	Protein coded by the gene
genes involved in tryptophan biosynthesis and IAA metabolism		
Q426_GM002478	*trpA*	tryptophan synthase subunit alpha
Q426_GM002479	*trpB*	tryptophan synthase subunit beta
Q426_GM002481	*trpC*	indole-3-glycerol phosphate synthase
Q426_GM002483	*trpE*	anthranilate synthase component I
Q426_GM000820	*dhaS*	aldehyde dehydrogenase
Q426_GM000792	*ysnE*	N-acetyltransferase
Q426_GM001039	*yhcX*	putative amidohydrolase
genes involved in the production of VOCs		
Q426_GM004101	*alsD*	alpha-acetolactate decarboxylase
Q426_GM004102	*ilvB*	alpha-acetolactate synthase
Q426_GM000711	*dbhA*	(R,R)-butanediol dehydrogenase
Q426_GM003379	*acuC*	acetoin dehydrogenase
genes involved in biofilm formation, development, and regulation		
Q426_GM001965	*ymcA*	cell fate regulator YmcA
Q426_GM003809	*iolU*	scyllo-inositol 2-dehydrogenase NADP( +)
Q426_GM000004	*slrR*	regulator of the extracellular matrix
genes involved in the synthesis of lipopeptides		
Q426_GM000375	*srfAA*	surfactin family lipopeptide synthetase A
Q426_GM000379	*srfAB*	surfactin family lipopeptide synthetase B
Q426_GM000380	*srfAC*	surfactin family lipopeptide synthetase C
Q426_GM000383	*srfAD*	surfactin family lipopeptide synthetase D
Q426_GM002145	*ituA*	iturin family lipopeptide synthetase A
Q426_GM002143	*ituB*	iturin family lipopeptide synthetase B
Q426_GM002139	*ituC*	iturin family lipopeptide synthetase C
Q426_GM002146	*ituD*	iturin family lipopeptide synthetase D
Q426_GM002177	*fenA*	fengycin family lipopeptide synthetase A
Q426_GM002176	*fenB*	fengycin family lipopeptide synthetase B
Q426_GM002182	*fenC*	fengycin family lipopeptide synthetase C
Q426_GM002170	*fenD*	fengycin family lipopeptide synthetase D
Q426_GM002179	*fenE*	fengycin family lipopeptide synthetase E

**Fig. 11:**
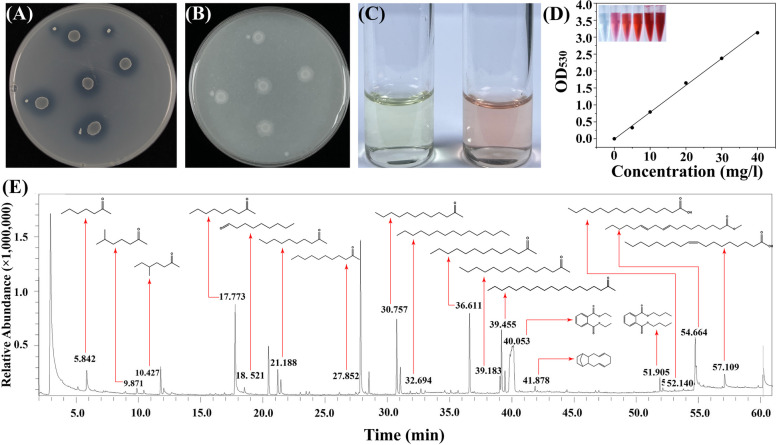
Plant growth-promoting activity test of *B. velezensis* Q-426. **A** Organic phosphate solubilization activity. **B** Inorganic phosphate solubilization activity. **C** Pink chromogenic reaction of IAA produced in *B. velezensis* Q-426. **D** Standard curve of IAA. **E** GC/MS of VOCs produced by *B. velezensis* Q-426

To verify the phosphate-solubilizing ability of Q-426, we performed the test using the hydrolysis of organic and inorganic phosphorus medium. We observed a clear transparent circle around the colony, which proved that Q-426 had organic phosphorolytic activity (Fig. 11A) but did not have inorganic phosphorolytic activity (Fig. 11B). IAA production in *B. velezensis* Q-426 was determined qualitatively and quantitatively. Q-426 was confirmed to produce IAA through the pink chromogenic reaction, and IAA production seemed to be high according to the pink color (Fig. 11C). The IAA production of Q-426 was further quantified by the calculated standard curve Equation y = 0.07933x-0.0105 (*R*^2^ = 0.999, y is the absorbance and x is IAA concentration). Q-426 showed the maximum IAA content of 1.56 mg/l on the third day of incubation (Fig. 11D). These results suggest that *B. velezensis* Q-426 could produce IAA even in the absence of exogenous tryptophan.

The genome mining results suggested that there were several genes involved in the production of VOCs. Then, we collected the VOCs produced by Q-426 on LB medium and analyzed them by GC/MC. Eighteen VOCs with relatively high peak areas were identified from Q-426. The retention times ranged from 1 to 60 min, with molecular weights ranging from 114.19 to 294.48 Da (Fig. 11E, Table 5). The identified VOCs included ten ketones (2-heptanone, 6-methylheptan-2-one, 5-methylheptan-2-one, 2-nonanone, 2-decanone, 2-undecanone, 2-dodecanone, 2-tridecanone, 2-tetradecanone, and 2-nonadecanone) and two acids (pentadecanoic acid and oleic acid). In addition, three esters (dethyl phthalate, dibutyl phthalate, and methyl (9E,12E)-octadeca-9,12-dienoate), two hydrocarbons (pentadecane, and (6E,10E)-1,2,3,4,4a,5,8,9,12,12a-decahydro-1,4-methanobenzo[10]annulene), and one aldehyde (nonanal) were present in the samples collected from Q-426. 2-heptanone displayed the highest peak of the 11 compounds detected in the VOC spectrum emitted by Q-426. Jiang et al. had reported that VOCs produced by *Bacillus sp.* JCo3 could significantly promote the biomass accumulation of Arabidopsis and tomato [46]. They identified that the compounds released from JC03, including 2-heptanone, tetrahydrofuran-3-ol and 2-ethyl-1-hexanol, significantly promoted the growth of Arabidopsis seedlings [46]. Widada et al. found that two known antifungal compounds 1,2-dimethyldisulfane and 6-methylheptan-2-one, were produced by *Nocardiopsis alba*, and the abundant of antifungal VOCs are potentially used as biocontrol agents for pathogenic fungi in plants [47]. Héctor et al. reported that the VOCs produced by *B. amyloliquefaciens* BUZ-14 and I3 have antifungal activity, and 2-nonanone and 2-undecanone have shown low concentrations for in vitro inhibition of *Monilinia spp.* and *B. cinerea* [48]. Li et al. found that nonanal, octanal and decanal could effectively inhibit the growth of *Aspergillus. flavus* both on maize kernels and in culture medium, these results confirmed that the plant-derived compounds could be developed into promising antifungal agents applied in the preservation of grains [49]. The 2-decanone was found in the volatilomes of three *Bacillus* species and showed antifungal activity [50–54]. 2-Nonadecanone is a ketone that showed antibacterial activity against *Staphylococcus aureus* and *Escherichia coli* [55]. The GC/MS results suggested that the antifungal and antimicrobial activity of VOCs can play important roles over short and long distances in promoting the growth of plants.

**Table 5:** The identification of VOCs produced by Q-426

Ret. Time	Area%	Height%	Name	Chemical Formula	Function
5.842	1.60	1.56	2-Heptanone	C_7_H_14_O	promote plant growth [46]
9.871	0.37	0.44	6-methylheptan-2-one	C_8_H_16_O	antifungal [47]
10.427	0.21	0.28	5-methylheptan-2-one	C_8_H_16_O	___
17.773	6.56	7.24	2-Nonanone	C_9_H_18_O	antifungal [48]
18.521	0.30	0.42	Nonanal	C_9_H_18_O	antifungal [49]
21.188	1.54	2.01	2-Decanone	C_10_H_20_O	antimicrobial [50–54]
27.852	9.91	12.53	2-Undecanone	C_11_H_22_O	repellant [56]
30.757	4.80	6.13	2-Dodecanone	C_12_H_24_O	repellant and repellant and insecticide [57]
32.694	0.24	0.35	Pentadecane	C_15_H_32_	bacteriostatic [58]
36.611	4.79	6.53	2-Tridecanone	C_13_H_26_O	repellant and antimicrobial [59]
39.183	3.75	5.11	2-Tetradecanone	C_15_H_26_	antifungal [60]
39.455	1.27	1.77	2-Nonadecanone	C_14_H_28_O	antimicrobial [55]
40.053	3.39	3.71	Diethyl Phthalate	C_19_H_38_O	___
41.878	0.39	0.45	Annulene^a^	C_12_H_14_O_4_	___
51.905	0.54	1.18	Dibutyl phthalate	C_15_H_22_	___
52.140	0.17	0.27	Pentadecanoic acid	C_16_H_22_O_4_	___
54.664	0.28	0.50	Methyl (9E,12E)-octadeca-9,12-dienoate	C_15_H_30_O_2_	___
57.109	0.90	1.06	Oleic Acid	C_19_H_34_O_2_	___

^a^annulene: (6E,10E)-1,2,3,4,4a,5,8,9,12,12a-decahydro-1,4-methanobenzo[10]annulene

## Conclusions

To explore the insights into the genetic characteristics and potential application of *B. velezensis* Q-426, in this study, we performed genome sequencing, gene functional annotation, and secondary metabolite biosynthetic gene cluster prediction of Q-426, which was isolated from compost samples. The Q-426 strain had high degree of collinearity with *B. velezensis* FZB42, *B. velezensis* SQR9, and *B. amyloliquefaciens* DSM7, and the strain was reidentified as *B. velezensis* Q-426 based on the phylogenetic tree. There are many genes in the Q-426 genome basis for plant growth-promoting activity, including the secondary metabolites of lipopeptides. Genome mining revealed 14 clusters and 732 genes encoding secondary metabolites with predicted functions, including surfactin, iturin and fengycin families. Twelve kinds of lipopeptides (surfactin, iturin and fengycin) were successfully detected from the fermentation broth of *B. velezensis* Q-426 by ultra-high performance liquid chromatography-quadrupole time-of-flight mass spectrometry (UHPLC–QTOF–MS/MS), which matched the genome analysis. We analyzed the potential surfactin gene clusters using antiSMASH, and the results suggested that the amino acid sequence might be composed of Glu/Asp-Leu/Ile/Val-Leu-Val-Asp-Glu/Asp-Leu/Ile/Val. We found 36 kinds of potential surfactins, and 16 of them had different molecular weights. We also found that Q-426 produced IAA and VOCs, which might promote the growth of plants. In conclusion, we mined secondary metabolite-related genes from the genome based on the whole genome sequence results. Our study laid the theoretical foundation for the development of secondary metabolites and the application of *B. velezensis* Q-426.

## Methods

### Bacterial strains and genomic DNA preparation

*B. velezensis* Q-426 was isolated from compost samples and stored in our laboratory (Key Laboratory of Biotechnology and Bioresources Utilization of Ministry of Education, Dalian Minzu University). Q-426’s whole genome was sequenced and submitted to GenBank (accession number CP102351). Q-426 was cultured in LB at 310 K for 20 h. Then the cells were harvested by centrifugation at 13,000 rpm for 30 min at 277 K. The cell pellet was collected and used to extract genomic DNA with a Bacterial DNA Isolation Kit (FOREGENE, China). The quality of DNA was assessed using agarose gel electrophoresis. The absorbances at 260 and 280 nm (A_260/280_) were estimated to check the quality and quantity of the extracted DNA sample.

### Genome sequencing and assembly

Genomic DNA was extracted with the SDS method. The harvested genomic DNA was detected by the agarose gel electrophoresis and quantified by Qubit® 2.0 Fluorometer (Thermo Scientific). The genome of Q-426 was sequenced by Single Molecule Real-Time (SMRT) technology. Sequencing was performed at the Beijing Novogene Bioinformatics Technology Co., Ltd. The low-quality reads were filtered by the SMRT Link v5.0.1 and the filtered reads were assemblied to generate one contig without gaps.

### Genome component prediction

Genome component prediction included the prediction of the coding gene, repetitive sequences, noncoding RNA, genomics islands, transposon, prophage, and clustered regularly interspaced short palindromic repeat sequences (CRISPR). We used the GeneMarkS program to retrieve the related coding genes. The interspersed repetitive sequences were predicted using RepeatMasker (http://www.repeatmasker.org/). The tandem repeats were analyzed by the TRF (Tandem repeats finder). Transfer RNA (tRNA) genes were predicted by tRNAscan-SE. Ribosome RNA (rRNA) genes were analyzed by rRNAmmer. Small nuclear RNAs (snRNA) were predicted by BLAST against the Rfam database. The IslandPath-DIOMB program was used to predict the genomics islands, and transposon PSI was used to predict the transposons based on the homologous blast method. PHAST was used for prophage prediction (http://phast.wishartlab.com/), and CRISPRFinder was used for CRISPR identification.

### Genome functional annotation and analysis

We used seven databases to predict gene functions. They were respective GO (Gene Ontology) [26], KEGG (Kyoto Encyclopedia of Genes and Genomes) [28], COG (Clusters of Orthologous Groups) [24], NR (Non-Redundant Protein Database databases) [61], TCDB (Transporter Classification Database), and Swiss-Prot. A whole genome Blast search (E-value less than 1e-5, minimal alignment length percentage larger than 40%) was performed against above seven databases. Genome overview was created by Circos to show the annotation information. The phylogenetic tree was constructed by the TreeBeST [62] or PhyML and the setting of bootstraps was 1,000 with the orthologous genes.

### Comparative genomics

Genomic alignment between the sample genome and reference genome was performed using MUMmer. Genomic synteny was analyzed based on the alignment results. For the comparison between the genome of *B. velezensis* Q-426 and other *Bacillus* strains (including *B. velezensis* FZB42 [6], *B. amyloliquefaciens* DSM7 [37], *B. velezensis* SQR9 [7], and *B. subtilis* 168 [38]), a circular comparative genome map was constructed using BLAST Ring Image Generator (BRIG) [63]. The NCBI GenBank server was used to download the genome of all strains.

### Secondary metabolite analysis and identification

AntiSMASH 4.0 software [41] was used to identify the gene clusters related to secondary metabolites. Using antiSMASH 4.0 with the default settings, the structure of secondary metabolism was detected against MIBiG. The lipopeptides in *B. velezensis* Q-426 were cultured and analyzed by LC–MS/MS according to a previously described method [64]. The *B. velezensis* Q-426 strain was cultured in bran medium (100 g bran/L, 20 g dextrose/L, 15 g agar/L) for three days at 28 ℃. The extracts were dissolved in 150 μL methanol and centrifuged at 15,000 × g for 20 min. The supernatants were transferred into HPLC autosampler vials and analyzed on an LTQ Orbitrap XL mass spectrometer (Thermo Fisher Scientific, Hemel Hempstead, UK) at a flow rate of 0.6 ml/min. Mass scanning range was m/z 550–1,500 Da in centroid mode with a scan rate of 1.5 spectra/s. The mass detection was performed by an electrospray source functioning in positive and negative ion modes at 15,000 resolving power. The mass measurement was externally calibrated before the experiment. Each full MS scan was followed by data-dependent MS/MS on the three most intense peaks using stepped collision-induced dissociation (35% normalized collision energy, isolation width 2 Da, activation Q 0.250).

### Identification of the plant growth-promoting activity of Q-426

The qualitative assay of IAA production was determined by the method described by Zhou, et al. [14]. *B. velezensis* Q-426 was inoculated in LB medium and incubated in a shaker (180 rpm, 37 ℃) for 2 days. Two milliliters of the culture was mixed with 2 ml of Salkowski reagent (50 ml 35% HClO_4_ + 1 ml 0.5 M FeCl_3_), and incubated for 30 min at 40 ℃ in dark. The appearance of pink color indicates IAA production. The noninoculated broth medium was used as a control. All treatments consisted of three replicates and were repeated twice. For the quantitative assay of IAA production, 2 ml of Q-426 supernatant was mixed with 2 ml of Salkowski’s reagent. The optical density of the sample was measured at 530 nm and the amount of Q-426 IAA produced was calculated by comparison with the standard IAA curve.

To verify whether Q-426 is a phosphate-solubilizing bacterium, we performed a test using the hydrolysis of organic and inorganic phosphorus medium. The seed solution of Q-426 was inoculated in LB medium and incubated in a shaker (180 rpm, 37 ℃) for 12 h. Single colonies were marked on the corresponding medium with seed solution, incubated in an incubator at 37 ℃ for 24 h. Then, the transparent circle around the colony was observed.

The VOCs produced by Q-426 were identified using a gas chromatograph-mass spectrometer (GC/MS, QP2010 Plus, Shinadzu). Q-426 was inoculated in LB medium and incubated in a shaker (180 rpm, 37 ℃) for 2 days. The rotor wrap was placed to seal, the extraction needle was inserted, and the extraction was stired at room temperature for 4 h. The results of detection were carried out by matching the molecular weights and fragmentation patterns of isolated compounds with compounds in the GC/MS library. By using GC/MS, the compounds produced can be used to determine the molecular weight and molecular structure of these compounds.

## Data Availability

The genome of strain Q-426 was deposited at NCBI under the GenBank accession number CP102351. The genomes of *B. velezensis* FZB42, *B. velezensis* SQR9, *B. amyloliquefaciens* DSM7 and *B. sublitis* 168 were download at NCBI under the GenBank accession number CP000560, CP006890, GCA_000196735, and CP051680, respectively.

## References

[1] Giomi T, Runhaar P, Runhaar H (2018). Reducing agrochemical use for nature conservation by Italian olive farmers: an evaluation of public and private governance strategies. Int J Agric Sustain.

[2] Pirttila AM, Mohammad ParastTabas H, Baruah N, Koskimaki JJ (2021). Biofertilizers and biocontrol agents for agriculture: how to identify and develop new potent microbial strains and traits. Microorganisms.

[3] Pii Y, Mimmo T, Tomasi N, Terzano R, Cesco S, Crecchio C (2015). Microbial interactions in the rhizosphere: beneficial influences of plant growth-promoting rhizobacteria on nutrient acquisition process. A review. Biol Fertil Soils.

[4] Fira D, Dimkic I, Beric T, Lozo J, Stankovic S (2018). Biological control of plant pathogens by Bacillus species. J Biotechnol.

[5] Reva ON, Swanevelder DZH, Mwita LA, Mwakilili AD, Muzondiwa D, Joubert M, Chan WY, Lutz S, Ahrens CH, Avdeeva LV (2019). Genetic, epigenetic and phenotypic diversity of four bacillus Velezensis strains used for plant protection or as probiotics. Front Microbiol.

[6] Fan B, Wang C, Song X, Ding X, Wu L, Wu H, Gao X, Borriss R (2018). Bacillus velezensis FZB42 in 2018: the gram-positive model strain for plant growth promotion and biocontrol. Front Microbiol.

[7] Sun X, Xu Z, Xie J, Hesselberg-Thomsen V, Tan T, Zheng D, Strube ML, Dragos A, Shen Q, Zhang R (2022). Bacillus velezensis stimulates resident rhizosphere Pseudomonas stutzeri for plant health through metabolic interactions. ISME J.

[8] Abd El-Daim IA, Bejai S, Fridborg I, Meijer J (2018). Identifying potential molecular factors involved in Bacillus amyloliquefaciens 5113 mediated abiotic stress tolerance in wheat. Plant Biol (Stuttg).

[9] Chen XH, Koumoutsi A, Scholz R, Eisenreich A, Schneider K, Heinemeyer I, Morgenstern B, Voss B, Hess WR, Reva O (2007). Comparative analysis of the complete genome sequence of the plant growth-promoting bacterium Bacillus amyloliquefaciens FZB42. Nat Biotechnol.

[10] Erlacher A, Cardinale M, Grosch R, Grube M, Berg G (2014). The impact of the pathogen Rhizoctonia solani and its beneficial counterpart Bacillus amyloliquefaciens on the indigenous lettuce microbiome. Front Microbiol.

[11] Chowdhury SP, Hartmann A, Gao X, Borriss R (2015). Biocontrol mechanism by root-associated Bacillus amyloliquefaciens FZB42 - a review. Front Microbiol.

[12] Xu Z, Shao J, Li B, Yan X, Shen Q, Zhang R (2013). Contribution of bacillomycin D in Bacillus amyloliquefaciens SQR9 to antifungal activity and biofilm formation. Appl Environ Microbiol.

[13] Chen L, Liu Y, Wu G, Veronican Njeri K, Shen Q, Zhang N, Zhang R (2016). Induced maize salt tolerance by rhizosphere inoculation of Bacillus amyloliquefaciens SQR9. Physiol Plant.

[14] Zhou H, Ren ZH, Zu X, Yu XY, Zhu HJ, Li XJ, Zhong J, Liu EM (2021). Efficacy of plant growth-promoting bacteria bacillus cereus YN917 for biocontrol of rice blast. Front Microbiol.

[15] Wang JH, Yang CY, Fang ST, Lu J, Quan CS (2016). Inhibition of biofilm in Bacillus amyloliquefaciens Q-426 by diketopiperazines. World J Microbiol Biotechnol.

[16] Zhao P, Quan C, Jin L, Wang L, Guo X, Fan S (2013). Sequence characterization and computational analysis of the non-ribosomal peptide synthetases controlling biosynthesis of lipopeptides, fengycins and bacillomycin D, from Bacillus amyloliquefaciens Q-426. Biotech Lett.

[17] Zhao P, Quan C, Jin L, Wang L, Wang J, Fan S (2013). Effects of critical medium components on the production of antifungal lipopeptides from Bacillus amyloliquefaciens Q-426 exhibiting excellent biosurfactant properties. World J Microbiol Biotechnol.

[18] Zhao P, Quan C, Wang Y, Wang J, Fan S (2014). Bacillus amyloliquefaciens Q-426 as a potential biocontrol agent against Fusarium oxysporum f. sp. spinaciae. J Basic Microbiol.

[19] Zhao J, Zhao P, Quan C, Jin L, Zheng W, Fan S (2015). Comparative proteomic analysis of antagonistic Bacillus amyloliquefaciens Q-426 cultivated under different pH conditions. Biotechnol Appl Biochem.

[20] Shi X, Liu J, Dong J, Wu Y, Quan C (2019). Bioinformatic analysis of two-component signal transduction systems in Bacillus amyloliquefaciens Q426. Biotechnol..

[21] Quan C, Jin L, Zhou W, Liu J, Shi X, Zheng W, Liu J, Fan R, Zhang L, Zhao P et al. The ComQXPA Quorum Sensing System May Play an Important Role in the Synthesis of Bacillomycin D in Bacillus Amyloliquefaciens Q-426. BMC microbiolo. 2020:1–26.

[22] Tao S, Zheng W, Zhao P, Zhou W, Quan C, Fan S (2014). Effects of bmy Gene knockout on Hemolysis and Antifungal activity of Bacillus amyloliquefaciens Q-426. China Biotechnol..

[23] Quan C, Liu J, Zhou W, Zheng W, Jin L, Zhao J, Zhao P, Fan S (2018). Isolation, purification and antitumor activity of Bacillomycin D from Bacillus amyloliquefaciems Q-426. Chin J Biotechnol.

[24] Galperin MY, Wolf YI, Makarova KS, Vera Alvarez R, Landsman D, Koonin EV (2021). COG database update: focus on microbial diversity, model organisms, and widespread pathogens. Nucleic Acids Res.

[25] Blum M, Chang HY, Chuguransky S, Grego T, Kandasaamy S, Mitchell A, Nuka G, Paysan-Lafosse T, Qureshi M, Raj S (2021). The InterPro protein families and domains database: 20 years on. Nucleic Acids Res.

[26] Ashburner M, Ball CA, Blake JA, Botstein D, Butler H, Cherry JM, Davis AP, Dolinski K, Dwight SS, Eppig JT (2000). Gene ontology: tool for the unification of biology The Gene Ontology Consortium. Nat Genet.

[27] Araujo FA, Barh D, Silva A, Guimaraes L, Ramos RTJ (2018). GO FEAT: a rapid web-based functional annotation tool for genomic and transcriptomic data. Sci Rep.

[28] Kanehisa M, Goto S (2000). KEGG: kyoto encyclopedia of genes and genomes. Nucleic Acids Res.

[29] Voichek M, Maass S, Kroniger T, Becher D, Sorek R (2020). Peptide-based quorum sensing systems in Paenibacillus polymyxa. Life Sci Alliance.

[30] Preda VG, Sandulescu O (2019). Communication is the key: biofilms, quorum sensing, formation and prevention. Discoveries (Craiova).

[31] Huynh T, Voros M, Kedves O, Turbat A, Sipos G, Leitgeb B, Kredics L, Vagvolgyi C, Szekeres A (2022). Discrimination between the two closely related species of the operational group. B amyloliquefaciens based on whole-cell fatty acid profiling. Microorganisms.

[32] Borriss R, Chen XH, Rueckert C, Blom J, Becker A, Baumgarth B, Fan B, Pukall R, Schumann P, Sproer C (2011). Relationship of Bacillus amyloliquefaciens clades associated with strains DSM 7T and FZB42T: a proposal for Bacillus amyloliquefaciens subsp. amyloliquefaciens subsp. nov. and Bacillus amyloliquefaciens subsp. plantarum subsp. nov. based on complete genome sequence comparisons. Int J Syst Evol Microbio.

[33] Dunlap CA, Saunders LP, Schisler DA, Leathers TD, Naeem N, Cohan FM, Rooney AP (2016). Bacillus nakamurai sp. nov., a black-pigment-producing strain. Int J Syst Evol Microbiol.

[34] Wang LT, Lee FL, Tai CJ, Kuo HP (2008). Bacillus velezensis is a later heterotypic synonym of Bacillus amyloliquefaciens. Int J Syst Evol Microbiol.

[35] Dunlap CA, Kim SJ, Kwon SW, Rooney AP (2016). Bacillus velezensis is not a later heterotypic synonym of Bacillus amyloliquefaciens; Bacillus methylotrophicus, Bacillus amyloliquefaciens subsp. plantarum and 'Bacillus oryzicola' are later heterotypic synonyms of Bacillus velezensis based on phylogenomics. Int J Syst Evol Microbiol.

[36] Mowafy AM, Fawzy MM, Gebreil A, Elsayed A (2021). Endophytic Bacillus, Enterobacter, and Klebsiella enhance the growth and yield of maize. Acta Agric Scand B Soil Plant Sci.

[37] Ruckert C, Blom J, Chen X, Reva O, Borriss R (2011). Genome sequence of B. amyloliquefaciens type strain DSM7(T) reveals differences to plant-associated B. amyloliquefaciens FZB42. J Biotechnol.

[38] Barbe V, Cruveiller S, Kunst F, Lenoble P, Meurice G, Sekowska A, Vallenet D, Wang T, Moszer I, Medigue C (2009). From a consortium sequence to a unified sequence: the Bacillus subtilis 168 reference genome a decade later. Microbiology.

[39] Tang H, Bowers JE, Wang X, Ming R, Alam M, Paterson AH (2008). Synteny and collinearity in plant genomes. Science.

[40] Xu Y, Bi C, Wu G, Wei S, Dai X, Yin T, Ye N (2016). VGSC: A web-based vector graph toolkit of genome Synteny and collinearity. Biomed Res Int.

[41] Blin K, Wolf T, Chevrette MG, Lu X, Schwalen CJ, Kautsar SA, Suarez Duran HG, de Los Santos ELC, Kim HU, Nave M (2017). antiSMASH 4.0-improvements in chemistry prediction and gene cluster boundary identification. Nucleic Acids Res.

[42] Pecci Y, Rivardo F, Martinotti MG, Allegrone G (2010). LC/ESI-MS/MS characterisation of lipopeptide biosurfactants produced by the Bacillus licheniformis V9T14 strain. J Mass Spectrom.

[43] Oviano M, Bou G (2019). Matrix-Assisted Laser Desorption Ionization-Time of Flight Mass Spectrometry for the Rapid Detection of Antimicrobial Resistance Mechanisms and Beyond. Clin Microbiol Rev..

[44] Santoyo G (2012). Orozco-Mosqueda MdC, Govindappa M: Mechanisms of biocontrol and plant growth-promoting activity in soil bacterial species of Bacillus and Pseudomonas: a review. Biocontrol Sci Tech.

[45] Upadhyay SK, Rajput VD, Kumari A, Espinosa-Saiz D, Menendez E, Minkina T, et al. Plant growth-promoting rhizobacteria: a potential bio-asset for restoration of degraded soil and crop productivity with sustainable emerging techniques. Environ Geochem Health. 2022. 10.1007/s10653-022-01433-3. Epub ahead of print. 10.1007/s10653-022-01433-3 36413266

[46] Jiang CH, Xie YS, Zhu K, Wang N, Li ZJ, Yu GJ, Guo JH (2019). Volatile organic compounds emitted by Bacillus sp. JC03 promote plant growth through the action of auxin and strigolactone. Plant Growth Regul.

[47] Widada J, Damayanti E, Alhakim MR, Yuwono T, Mustofa M (2021). Two strains of airborne Nocardiopsis alba producing different volatile organic compounds (VOCs) as biofungicide for Ganoderma boninense. FEMS Microbiol Lett.

[48] Calvo H, Mendiara I, Arias E, Gracia AP, Blanco D, Venturini ME (2020). Antifungal activity of the volatile organic compounds produced by Bacillus velezensis strains against postharvest fungal pathogens. Postharvest Biol Technol.

[49] Li Q, Zhu X, Xie Y, Liang J (2021). Antifungal properties and mechanisms of three volatile aldehydes (octanal, nonanal and decanal) on Aspergillus flavus. Grain Oil Sci Technol.

[50] Che J, Liu B, Liu G, Chen Q, Lan J (2017). Volatile organic compounds produced by Lysinibacillus sp. FJAT-4748 possess antifungal activity against Colletotrichum acutatum. Biocontrol Sci Technol.

[51] Jayakumar V, Ramesh Sundar A, Viswanathan R (2021). Biocontrol of Colletotrichum falcatum with volatile metabolites produced by endophytic bacteria and profiling VOCs by headspace SPME coupled with GC–MS. Sugar Tech.

[52] Yuan J, Raza W, Shen Q, Huang Q. Antifungal activity of Bacillus amyloliquefaciens NJN-6 volatile compounds against Fusarium oxysporum f. sp. cubense. Appl Environ Microbiol. 2012;78(16):5942–44. 10.1128/AEM.01357-12 PMC3406121 22685147

[53] Zheng M, Shi J, Shi J, Wang Q, Li Y (2013). Antimicrobial effects of volatiles produced by two antagonistic Bacillus strains on the anthracnose pathogen in postharvest mangos. Biol Control.

[54] Lammers A, Zweers H, Sandfeld T, Bilde T, Garbeva P, Schramm A, Lalk M (2021). Antimicrobial compounds in the volatilome of social spider communities. Front Microbiol.

[55] Mihigo DS (2015). Preliminary GC-MS Profiling and Anti-bacterial activity Investigation of Ageratum conyzoides Linn. (Asteraceae). Int J Chem Aquat Sci.

[56] Yang L, Norris EJ, Jiang S, Bernier UR, Linthicum KJ, Bloomquist JR (2020). Reduced effectiveness of repellents in a pyrethroid-resistant strain of Aedes aegypti (Diptera: culicidae) and its correlation with olfactory sensitivity. Pest Manag Sci.

[57] Wang Y, Zhang LT, Feng YX, Guo SS, Pang X, Zhang D, Geng ZF, Du SS (2019). Insecticidal and repellent efficacy against stored-product insects of oxygenated monoterpenes and 2-dodecanone of the essential oil from Zanthoxylum planispinum var. dintanensis. Environ Sci Pollut Res Int.

[58] Firdaus M, Kartikaningsih H, Sulifah U (2019). Sargassum spp extract inhibits the growth of foodborne illness bacteria. AIP Conf Proc.

[59] Gu M, Xue Z, Lv S, Cai Y, Zhang L, Gao X (2022). Corynebacterium sp. 2-TD Mediated Toxicity of 2-Tridecanone to Helicoverpa armigera. Toxins (Basel).

[60] Yuan J, Raza W, Shen Q, Huang Q (2012). Antifungal activity of Bacillus amyloliquefaciens NJN-6 volatile compounds against Fusarium oxysporum f. sp. cubense. Appl Environ Microbiol.

[61] Wilke A, Harrison T, Wilkening J, Field D, Glass EM, Kyrpides N, Mavrommatis K, Meyer F (2012). The M5nr: a novel non-redundant database containing protein sequences and annotations from multiple sources and associated tools. BMC Bioinformatics.

[62] Vilella AJ, Severin J, Ureta-Vidal A, Heng L, Durbin R, Birney E (2009). EnsemblCompara GeneTrees: Complete, duplication-aware phylogenetic trees in vertebrates. Genome Res.

[63] Alikhan NF, Petty NK, Ben Zakour NL, Beatson SA (2011). BLAST Ring Image Generator (BRIG): simple prokaryote genome comparisons. BMC Genomics.

[64] Sun Y, Liu WC, Shi X, Zheng HZ, Zheng ZH, Lu XH, Xing Y, Ji K, Liu M, Dong YS (2021). Inducing secondary metabolite production of Aspergillus sydowii through microbial co-culture with Bacillus subtilis. Microb Cell Fact.

